# Ruptured Emphysematous Prostatic Abscess Caused by K1-ST23 Hypervirulent *Klebsiella pneumoniae* Presenting as Brain Abscesses: A Case Report and Literature Review

**DOI:** 10.3389/fmed.2021.768042

**Published:** 2022-01-03

**Authors:** Kensuke Konagaya, Hiroyuki Yamamoto, Tomoyuki Suda, Yusuke Tsuda, Jun Isogai, Hiroyuki Murayama, Yoshichika Arakawa, Hidemitsu Ogino

**Affiliations:** ^1^Department of Surgery, Narita-Tomisato Tokushukai Hospital, Chiba, Japan; ^2^Department of Cardiovascular Medicine, Narita-Tomisato Tokushukai Hospital, Chiba, Japan; ^3^Department of Bacteriology, Nagoya University Graduate School of Medicine, Aichi, Japan; ^4^Department of Radiology, Asahi General Hospital, Asahi, Japan

**Keywords:** *Klebsiella pneumoniae*, hypervirulent, EPA, brain abscess, K1-ST23, NGS

## Abstract

Emphysematous prostatic abscess (EPA) is an extremely rare but potentially fatal urinary tract infection (UTI). Here, we describe a case (a 69-year-old male with prediabetes) of ruptured EPA caused by a hypervirulent *Klebsiella pneumoniae* (hvKp) K1-ST23 strain, presenting as motor aphasia. Our patient presented with ruptured EPA concurrent with various severe systemic pyogenic complications (e.g., urethro-prostatic fistula, ascending UTIs, epididymal and scrotal abscesses, and liver, lung, and brain abscesses). Whole-body computed tomography (CT) and next-generation sequencing (NGS) were useful for the detection of ruptured EPA and its systemic complications, and for identification of K1-ST23 hvKp strains, respectively. Subsequently, the infections were successfully treated with aggressive antimicrobial therapy and multiple surgical procedures. This case highlights the significance of awareness of this rare entity, the clinical importance of CT for the early diagnosis of EPA and the detection of its systemic complications in view of hvKp being an important causative organism of severe community-acquired UTI, and the usefulness of NGS to identify hvKp strains.

## Introduction

Prostatic abscess is an uncommon sequela of urinary tract infection (UTI). In the post-antimicrobial era, the incidence is low at approximately 0.5% (1). Among them, emphysematous prostatic abscess (EPA), characterized by gas accumulation and abscess formation within the prostate gland, is an extremely rare but life-threatening condition if left untreated (2). However, an accurate diagnosis remains challenging because of the non-specific symptoms and signs.

## Case Description

**Figure 1:**
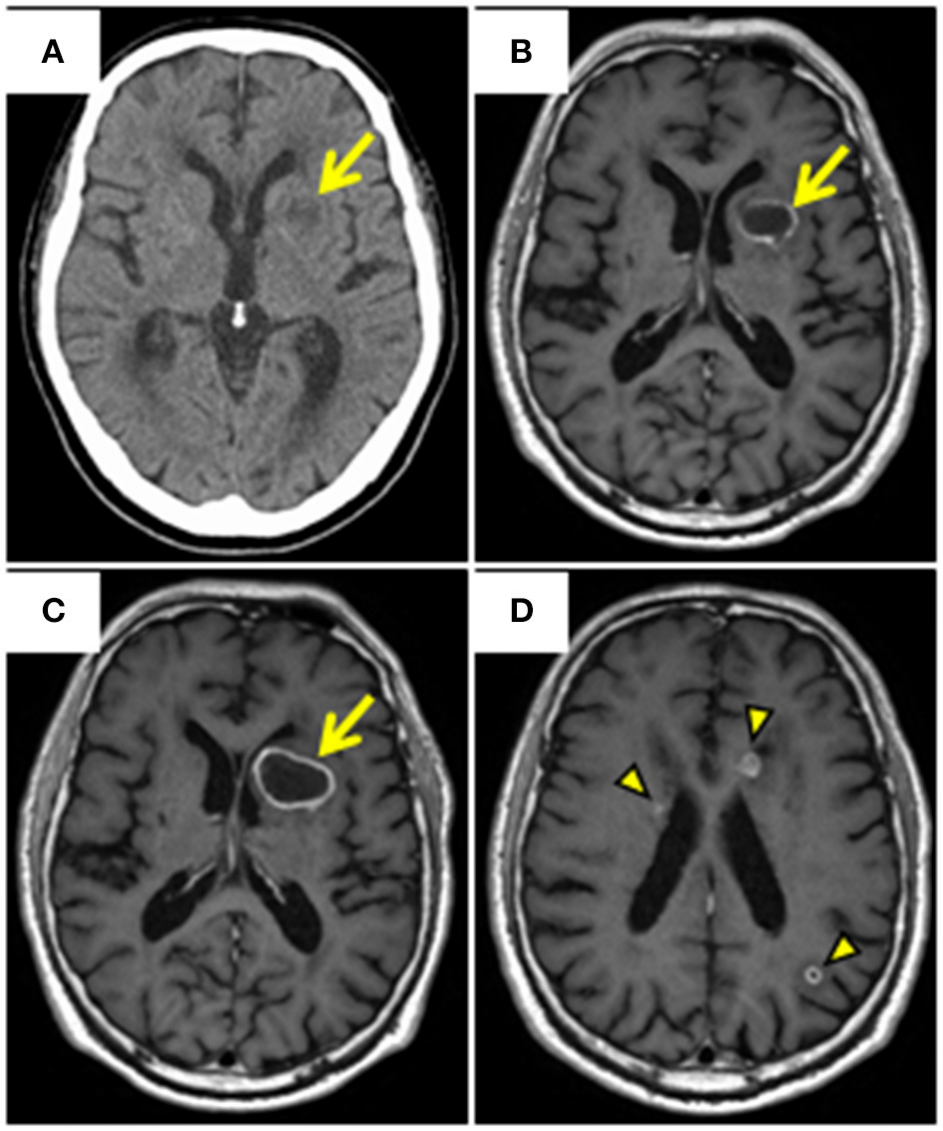
Brain CT and MRI. **(A)** Axial plain CT reveals a round low-attenuation area with slightly high-attenuation rim in the left lenticular nucleus (arrow). **(B)** Axial gadolinium-enhanced T1-weighted MRI reveals ring enhancement in the area (arrow). **(C, D)** MRI following the initial treatment reveals enlargement of the existing abscess (arrow), and new ring enhancement (arrowheads) in the bilateral periventricular and subcortical areas. CT, Computed tomography; MRI, Magnetic resonance imaging.

**Figure 2:**
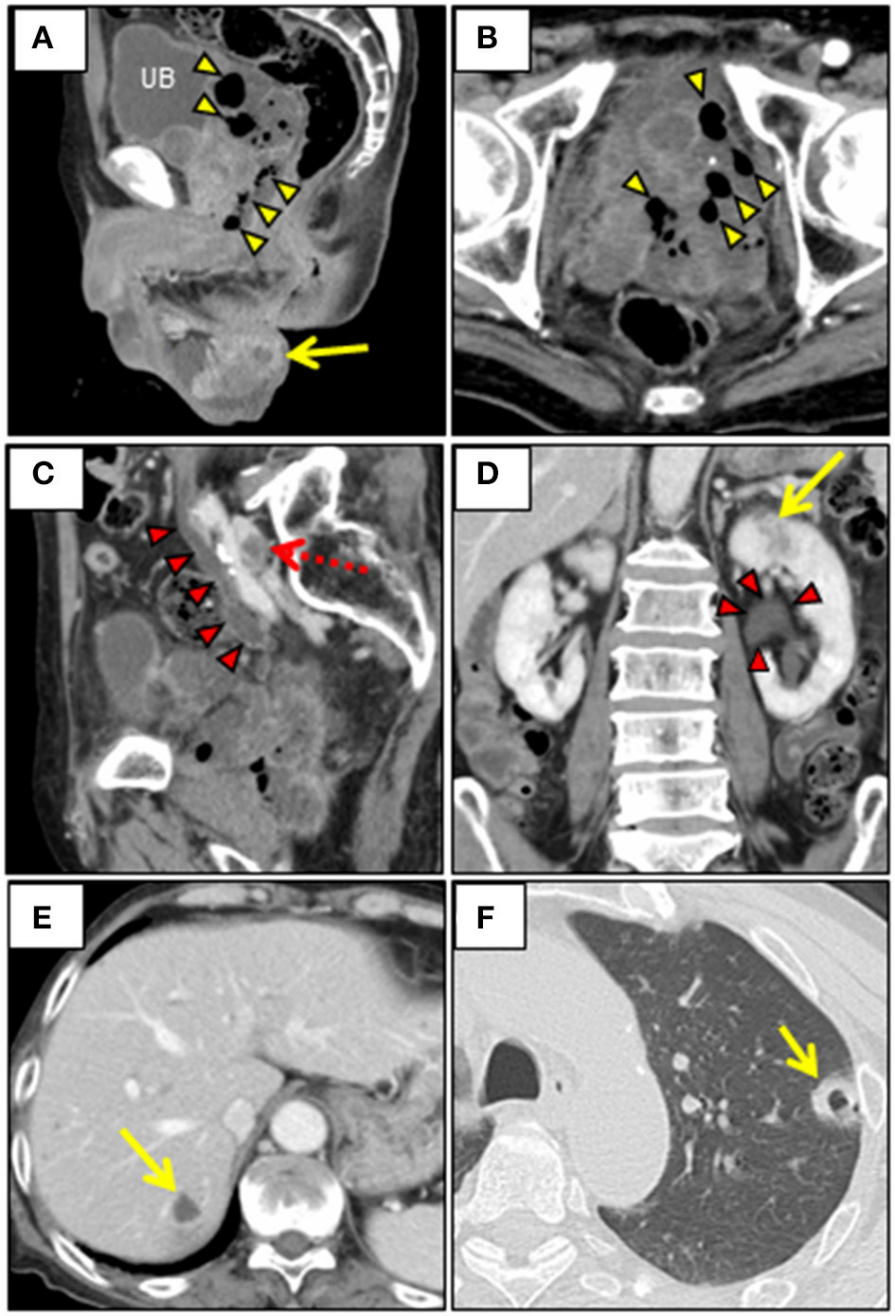
Contrast-enhanced whole-body CT. **(A, B)** Pelvic CT reveals the irregularly-shaped enlarged prostate and the left seminal vesicle, which are replaced by encapsulated fluid cavities containing multiple gas bubbles (arrowheads), with spread of infection into extraperitoneal perivesicular space in the small pelvis. Note the left epididymal abscess (arrow). **(C, D)** Abdominal/pelvic CT shows marked dilatation of the left ureteral and renal pelvis (arrowheads), and multiple hypoenhancement foci in the left renal parenchyma (arrow). Note the thrombus in the left common iliac vein parallel to the dilated ureter (dotted arrow). **(E)** Abdominal CT reveals a centrally hypoattenuating lesion with peripheral heterogeneous enhancement in liver S7 (arrow). **(F)** Chest CT (lung window) reveals peripheral thick-walled cavitary nodule in the left upper lobe (arrow). CT, Computed tomography; UB, Urinary bladder.

**Figure 3:**
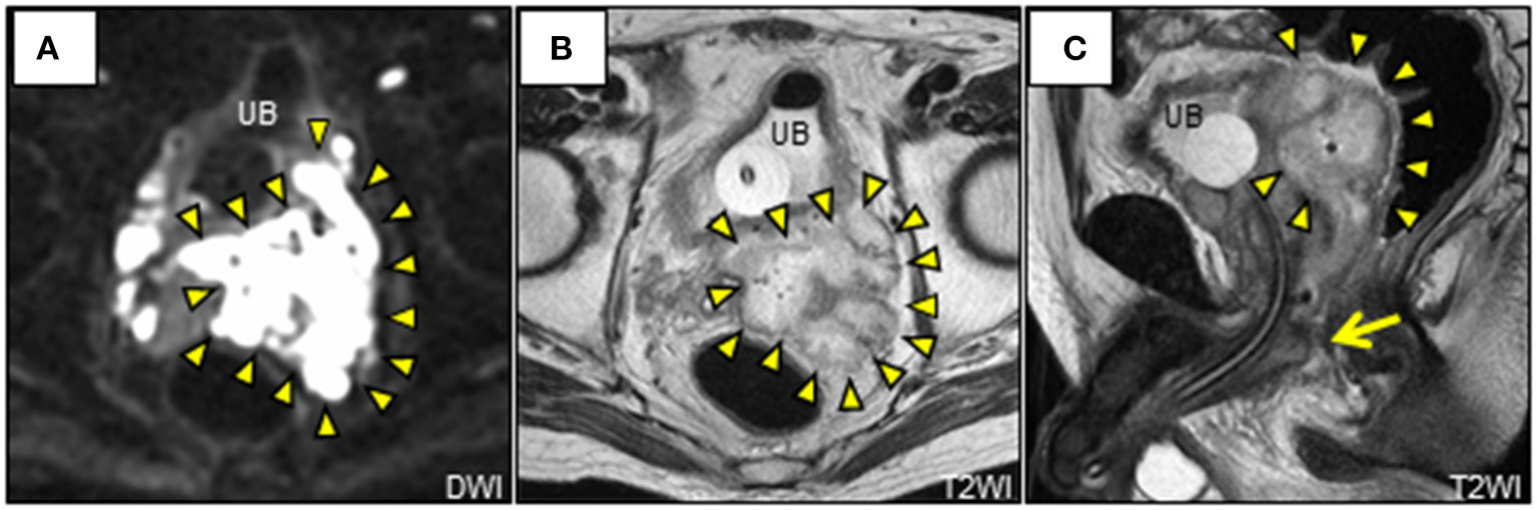
Pelvic magnetic resonance imaging. **(A)** Axial DWI shows characteristic restricted diffusion on the left lobe of the prostate extending to the opposite lobe and bilateral perivesicular extraperitoneal spaces (arrowheads). **(B, C)** Axial and sagittal T2WI shows obviously multilocular abscess of both prostate and left seminal vesicle (arrowheads), with a fistulous communication of the bulbar urethra (arrow). DWI, diffusion-weighted image; T2WI, T2-weighted image; UB, urinary bladder.

A 69-year-old Japanese man was admitted to our hospital with a 7-day history of slurred speech. He had no past history of prostatic manipulation. On admission, the patient had a low-grade fever of 37.8°C, tachycardia of 109 beats/min, blood pressure of 122/90 mmHg, and oxygen saturation of 97% on ambient air. Motor aphasia was recognized on neurologic examination. Digital rectal examination revealed a soft and non-tender enlarged prostate. Laboratory testing revealed neutrophilic leukocytosis, elevated levels of C-reactive protein (20.86 mg/dL; reference: <0.15 mg/dL), fasting blood glucose (135 mg/dL; reference: <110 mg/dL), and HbA1c (6.3%; reference: <6.0%). Mild hepatorenal dysfunction was also observed. Urinalysis was positive for bacteriuria and pyuria. Cerebrospinal fluid analysis was unremarkable. Brain computed tomography (CT) and magnetic resonance imaging (MRI) revealed a lesion in the left lenticular nucleus, suggestive of a brain abscess (Figures 1A,B). We subsequently searched for the cause of the brain abscess on a whole-body CT. Pelvic CT revealed an enlarged multilobular prostate gland characterized by capsulized fluid cavities and multiple gas bubbles, suggesting EPA caused by anaerobic bacteria (Figures 2A,B). We also recognized severe pyogenic complications of EPA, which were different from Fournier's gangrene. We classified a wide spectrum of complications into four predominant categories according to the route of spread of infection. We found direct extension as the 1st route, which involved spread into the left seminal vesicle and extraperitoneal space of the ischioanal fossa (Figures 2A,B), and an ascending infection of the ipsilateral urinary tract as the 2nd route, which caused left hydronephrosis, acute focal bacterial nephritis, and thrombophlebitis of the adjacent left iliac vein (Figures 2C,D). We also found a retrograde infection of the ipsilateral spermatic duct as the 3rd route, which led to an epididymal abscess (Figure 2A), and hematogenous spread to the liver (Figure 2E) and lungs (Figure 2F) as the 4th route. Pelvic MRI further characterized the pelvic infection and revealed active prostatic inflammation involving the urinary bladder and left seminal vesicles, which ruptured into the extraperitoneal perivesicular space, and subsequent fistula formation of the bulbar urethra with the prostate (Figure 3). Based on these findings, we concluded that brain abscess was highly likely due to hematogenous seeding of the primary prostate infection. We started to treat the pelvic infection with the insertion of a transurethral Foley catheter and commencement of empiric antimicrobial therapy (meropenem, 4 g/day IV).

On day 5, all the urine culture and two sets of blood cultures collected on admission yielded *Klebsiella pneumoniae*, which was susceptible to conventional antimicrobials, yet naturally resistant to ampicillin. Although the *K. pneumoniae* isolate was negative for the string test, we performed next-generation sequencing (NGS) for capsular genotyping, virulence factors, and multilocus sequencing typing (MLST). The isolate was found to belong to a sequence type (ST) 23 serotype K1 strain harboring virulence-associated genes, including *kfuA* (an iron uptake system)*, magA* (specific to K1 capsule serotype)*, rmpA* (regulator of mucoid phenotype), *ybtS* (yersiniabactin gene)*, iroN* (gene for the outer membrane receptor, FepA, of Fe^3+^-bound salmochelin), and *mrkD* (type 3 fimbrial adhesin gene). Based on the antimicrobial susceptibility test results, antimicrobial de-escalation was performed (ciprofloxacin, 1,200 mg/day IV). On day 14, a left scrotal abscess developed progressively, presumably originating from the adjacent epididymal abscess, and required surgical drainage. On day 21, follow-up CT showed a decrease in the size of the EPA, and multiple intrapelvic abscesses, liver abscess, and pulmonary septic emboli.

However, on day 24, we performed an additional brain abscess drainage on a CT-guided stereotactic procedure because follow-up brain MRI confirmed a gradual progression of the existing brain abscess and multiple new ones (Figures 1C,D). Subsequently, the treatment was replaced with a 3-week course of intravenous ceftriaxone (3 g/day) and ciprofloxacin (1,200 mg/day). On follow-up CT/MRI, the brain abscesses gradually reduced in size. The patient continued rehabilitation for disuse syndrome and motor aphasia, and these improved with training. Fasting blood glucose levels after control of infection were in the range of 110–125 mg/dL, suggesting prediabetes. The patient was discharged on day 88. He remained clinically stable at 1-year follow-up. The timeline of the case presentation and clinical course is presented in Supplementary Figure 1.

## Discussion

Herein, we describe a rare case of EPA caused by a hypervirulent *K. pneumoniae* (hvKp) K1-ST23 strain. Within a few days, the patient developed abscess rupture concurrent with various serious systemic pyogenic complications successfully treated with aggressive antimicrobial therapy and multiple surgical interventions. Our case illustrates several valuable clinical pearls.

First, our patient with EPA exhibited abscess rupture, leading to a variety of serious systemic pyogenic complications. We conducted an updated systematic review of case reports to investigate the characteristics and epidemiology of EPA (Table 1). The literature search was performed with English language restriction using electronic databases of PubMed from January 1, 1983, until July 28, 2021. The following search terms were used: “emphysematous prostatic abscess” (Mesh) or “emphysematous prostatic abscess” (tiab) or “emphysematous prostatitis” (Mesh) or “emphysematous prostatitis” (tiab). Consequently, 22 cases were included (2–21). EPA is most often diagnosed in Asia (68%), with an average age of 60 years (range, 35–75 years). The underlying disease was diabetes mellitus (96%), followed by liver cirrhosis (27%), recent urological surgery (23%), and alcoholism (18%). Unlike prostatic abscess, in which *Escherichia coli* is the main causative organism; the most common causative organism of EPA is *K. pneumoniae* (50%), followed by *Escherichia coli* (18%) and *Candida albicans* (14%). EPA is often misdiagnosed as the usual type of UTI, leading to time delay (range, 4–12 days) in the accurate diagnosis and proper treatment. EPA can have a variable presentation, ranging from asymptomatic to lethal. Frequent complications of EPA include localized spread of infection to adjacent areas, ascending infections, or disseminated hematogenous spread. Although rare, the most feared sequelae include a urethro-prostatic fistula or abscess rupture into the urethra, perineum, bladder, or rectum (13, 22). Metastatic infections are common (55%), with a high mortality rate of 23%. CT is the most useful diagnostic imaging tool. The mainstay of treatment includes adequate antimicrobial therapy and drainage; in 86% of cases, surgical drainage was performed using a transperineal (45%), transurethral (41%), or transrectal (9%) approach. Suprapubic cystostomy is often performed for urinary diversion (45.4%).

**Table 1:** Cases of EPA.

**References**	**Case**	**Country**	**Age**	**Underlying disease**	**Initial diagnosis**	**Time delays in the diagnosis of EPA**	**Metastatic infections**	**Diagnostic imaging**	**Cystostomy**	**Drainage**	**Pathogen**	**Culture**	**Outcome**
Mariani et al. (3)	1	USA	56	DM	UTI	N.A.	None	CT, IVP, Ga	Performed	TUIP	*P. aeruginosa*	Abscess, Blood, Urine	Survived
											*B. fragilis*	Abscess	
Bartkowski and Lanesky (4)	2	USA	60	DM, recurrent pancreatitis	UTI	10 days	EC	KUB, CT	Performed	TUIP	*C. albicans*	Blood, Urine	Survived
Lu et al. (5)	3	TWN	45	DM, Alcoholism	UTI with septic shock	4 days	EC	CT	Not performed	TPNA	*K. pneumoniae*	Blood, Pus	Died
Lin et al. (6)	4	TWN	55	DM, LC, HCC, s/p TUMT	EPA	None	Periurethral abscess	CT	Performed	DPID	*K. pneumoniae*	Abscess, Urine	Died
Bae et al. (7)	5	KOR	50	DM	EC	12 days	EC, Pyelonephritis	KUB, TRUS, CT	Performed	TPD	*K. pneumoniae*	Urine	Survived
Kuo et al. (2)	6	TWN	60	DM, Alcoholism, LC	EPA	None	None	KUB, TRUS, CT	Not performed	TUIP	*K. pneumoniae*	Blood	Survived
Sampathkumar et al. (8)	7	IND	57	DM, HTN, ESRD, s/p RTx	EPA	None	EC, EP	CT	Not performed	TUIP	*E.coli*	Blood, Urine	Died
Tai (9)	8	TWN	60	DM, Alcoholism, LC	EPA	None	None	KUB, TRUS, CT	Not performed	TUIP	*K. pneumoniae*	Blood, Urine	Survived
Juan et al. (10)	9	TWN	68	DM, LC	UTI with septic shock	7 days	None	CT	Not performed	CTPD	*C. albicans*	Pus, Urine	Survived
Thorner et al. (11)	10	USA	64	DM, ESRD	N.A.	N.A.	None	CT	Performed	TUIP	*Citrobacter sp*.	Urine	Survived
Cheung and Tsang (12)	11	TWN	68	DM, HTN, PU, Stroke	EPA	None	Scrotal abscess	CT	Not performed	TUIP	*E.coli*	Urine	Survived
Wen et al. (13)	12	TWN	72	DM, Alcoholism	Acute prostatitis	N.A.	None	KUB, TRUS, CT	Performed	CTPD	*K. pneumoniae*	Blood, Pus, Urine	Survived
	13	TWN	68	DM, LC	UTI with septic shock	7 days	None	CT	Not performed	CTPD	*C. albicans*	Pus, Urine	Survived
	14	TWN	81	s/p TUNA	EPA	None	Hydronephrosis	KUB, CT	Not performed	CTPD	*E.coli* (ESBL^+^)	Pus, Urine	Survived
							Prostatic fistula				*C.glabrata*	Pus, Urine	
Hsu et al. (14)	15	TWN	54	DM, LC	N.A.	N.A.	None	KUB, TRUS, CT	N.A.	TPNA and TUIP	*K. pneumoniae*	blood, urine, pus	Survived
											*E.coli*	Urine	
Lee et al. (15)	16	TWN	70	DM, s/p IUC insertion	EPA	None	EPUA	CT	Performed	CTPD	*K. pneumoniae*	Abscess, Blood, Urine	Survived
Wang and Shih (16)	17	TWN	42	DM	EPA	None	None	KUB, CT	Not performed	CTPD	*K. pneumoniae*	Blood, Urine	Survived
Suzuki et al. (17)	18	JPN	75	DM, AGC	EPA	None	EC, Renal abscess	KUB, CT	Not performed	None	K. pneumoniae^*^	Blood, Urine	Survived
Li et al. (18)	19	CHN	72	DM	UTI	8 days	None	CT	Performed	TRNA and TUIP	*C. tropicalis*	Urine	Survived
Metri et al. (19)	20	IND	60	DM, s/p TRUS-guided biopsy	EPA	None	Psoas and thigh abscess	CT	Not performed	None	*S. aureus*	Pus	Died
Mayouf et al. (20)	21	TUN	48	DM, CKD, Stroke	UTI with DKA	N.A.	None	CT	Performed	TRNA	*E. cloacae*	Blood, Urine	Died
Crane et al. (21)	22	USA	35	DM, LC	COVID-19 infection	12 days	Multiple organs^**^	TRUS	Performed	None	*K. pneumoniae*	Eye, Urine	Paropsia

*EPA, Emphysematous prostatic abscess; USA, United States; TWN, Taiwan; KOR, Korea; IND, India; JPN, Japan; CHN, China; TUN, Tunisia; DM, Diabetes; LC, Liver cirrhosis; HCC, Hepatocellular carcinoma; s/p, Status post; TUMT, Transurethral microwave thermotherapy; HTN, Hypertension; ESRD, End-stage renal disease; RTx, Renal transplantation; PU, Peptic ulcer; TUNA, Transurethral radiofrequency needle ablation; IUC, Indwelling urinary catheter; AGC, Advanced gastric cancer; TRUS, Transrectal ultrasonography; CKD, Chronic kidney disease; UTI, Urinary tract infection; EC, Emphysematous cystitis; N.A., Not Available; DKA, Diabetic ketoacidosis; EP, Emphysematous pyelitis; EPUA, Emphysematous periurethral abscess; CT, Computed tomography; IVP, Intravenous pyelogram; Ga, Gallium scan; KUB, The kidneys, ureters, bladder radiograph; TUIP, Transurethral incision of the prostate; TPNA, Transperineal needle aspiration; DPID, Direct perineal incision and drainage; TPD, Transperineal drainage; CTPD, Computed tomography-guided perineal drainage of the prostate; TRNA, Transrectal needle aspiration. ESBL^+^ denotes extended-spectrum beta-lactamase-producing; K. pneumoniae*

* *denotes hypervirulent strain (serotype K1); multiple organs*

** *denotes bilateral endophthalmitis, septic pulmonary emboli, penis, and bilateral internal iliac thrombosis*.

The present case presented with systemic complications via all the aforementioned routes of infection. Despite fatal complications (e.g., urethro-prostatic fistula, epididymal and scrotal abscesses, and metastatic hepatic/pulmonary/brain abscesses), CT/MRI screening was useful for early detection, and the infection was successfully controlled by aggressive antimicrobial therapy and multiple surgical drainage procedures.

Second, our case underscores the significance of early diagnosis of invasive *K. pneumoniae* infections in healthy subjects. The hvKp is a major causative agent in invasive community-acquired infections characterized by pyogenic liver abscess (PLA) with a high mortality, and has been reported in the Asian Pacific Rim, starting in Taiwan since the 1980s, and is now spreading worldwide (23). The hvKp strain has a much more vicious phenotype than the classical *K. pneumoniae* (cKp) strain due to its high toxicity and metastatic potential, resulting in grave secondary infections such as endophthalmitis, meningitis, and septic pulmonary emboli. Thus, early diagnosis and prompt treatment are required; however, the following three clinical issues for hvKp infection were exemplified by our case.

As the first clinical issue, hvKp-related primary extrahepatic genitourinary infections are increasingly being reported. Although the exact pathogenesis of hvKp remains unknown, many studies have indicated that hvKp initially colonizes the gastrointestinal tract, from where it preferentially infects other organs, mainly the liver. Therefore, UTI caused by hvKp has been considered secondary to hematogenous spread from preceding bacteremia. In fact, there are several reported cases of PLA with hematogenous spread to the kidney, perirenal tissues, and prostate, leading to local abscess formation (23). Conversely, several hvKp cases with community-acquired UTIs without PLA have also been reported (24–26). Similarly, the main source of infection in our case was the prostate gland, and concomitant liver abscess was highly likely secondary to the primary prostatic infection. Thus, our case underscores the importance of considering the possibility of hvKp infection even in patients with community-acquired UTIs.

The second clinical issue concerns the significance of serotype K1 *K. pneumoniae* in our case. To our knowledge, the present case is the first case of EPA caused by serotype K1 hvKp infection presenting with a brain abscess. The K1 strain has a strong propensity to cause metastatic ocular and neurologic complications. In a single-center, retrospective study of 177 patients with PLA in Taiwan, the incidence of metastatic complications was 13% (27). The frequency of metastatic complications was significantly higher in patients with K1 strains than in patients with non-K1 strains (19 vs. 5%, *p* = 0.007). Multivariate logistic regression analyses demonstrated that the K1 strain was the sole significant risk factor for metastatic complications (adjusted odds ratio, 4.8; 95% confidence interval, 1.5–15.7, *p* = 0.009), strongly suggesting that the K1 strains should be recognized as a distinct virulent organism. In addition, such metastatic complications have been reported in patients with K1 strains without hepatic involvement, suggesting that their development is unrelated to the presence of PLA (25, 26). Thus, our case highlights the importance of considering serotype K1 hvKp as a rare causative agent of community-acquired brain abscess and of screening for the primary site of infection.

The third clinical issue is to identify a hvKp strain definitely. Although numerous studies have been conducted to differentiate hvKp strains from cKp strains in terms of epidemiologic and genetic differences, there are still no clear microbiological and molecular diagnostic criteria for hvKp strains. A positive string test, indicating a hypermucoviscous colony phenotype on the agar plate, is widely believed to be a unique characteristic of hvKp strains. However, hypermucoviscosity and hypervirulence do not always coincide, as shown by the fact that there are both string test-negative hvKp strains and string test-positive cKp strains. A study evaluating 140 cases of *K. pneumoniae* bloodstream infections revealed that the string test alone is not adequate to differentiate hvKp strains from cKp strains (69.2% sensitivity, 89.5% specificity, 60.0% positive predictive value, and 92.7% negative predictive value), supporting this notion (28). The hvKp strains have some capsular types necessary for their virulence, with K1/K2 capsular serotypes being the most prevalent and virulent reported to date. Regarding virulence-associated genes, *rmpA* is remarkably related to the hvKp strain (29). Type 1 and type 3 fimbriae are deeply involved in bacterial resistance to phagocytosis, adhesion to biological/non-biological surfaces, and altered antibiotic permeability. Iron is a metal necessary for bacterial growth and plays an important role in infection spread, and siderophores (aerobactin, enterobactin, salmochelin, and yersiniabactin), secreted by *K. pneumoniae*, have the ability to sequester iron, contributing to their high virulence. However, while all the factors described above play important roles in the process of increased virulence, no single causal risk factor alone is the sole determinant of hypervirulence. Recently, a new genotype/phenotype approach for the comprehensive detection of a specific set of biomarkers has been proposed for the successful identification of hvKp strains (30). Further investigation is expected to accumulate in the future. In our case, NGS was useful for identifying the hvKp strain because it allows simultaneous analysis of MLST and virulence-associated genes, belongs to a K1-ST23 hvKp strain, and has the same profile of virulence-associated genes as the K1-ST23 hvKp strains previously identified in Japan, suggesting high genetic similarity (31). In our case review, only one case of EPA caused by the serotype K1 hvKp strain has been reported (Table 1). Given the high incidence of *K. pneumoniae* pathogens in EPA patients and the highly invasive nature of EPA, a thorough investigation of these strains is warranted.

Untreated glucose intolerance might have contributed to the virulent phenotype observed in our case, since hyperglycemia impairs neutrophil phagocytosis of K1/K2 capsular serotypes (32).

## Conclusions

Herein, we report a case of ruptured EPA secondary to K1-ST23 hvKp infection with serious systemic pyogenic complications that was successfully treated with aggressive antimicrobial therapy and multiple surgical procedures. CT is useful for the accurate diagnosis and early detection of complications. EPA is an extremely rare, yet potentially fatal sequela of UTI, which can be curable if treated properly at an early stage. Therefore, clinicians should recognize this rare clinical entity and perform systemic CT screening in patients with potentially severe UTI. In addition, our case highlights the clinical importance of keeping in mind hvKp infections as a possible cause of community-acquired UTI in immunocompromised individuals including prediabetes, and of NGS to identify hvKp strains for appropriate treatment.

## Data Availability Statement

The raw data supporting the conclusions of this article will be made available by the authors, without undue reservation.

## Author Contributions

HY was responsible for the clinical study design and conceptualization of the study. KK, TS, HM, HY, and HO were involved in the acquisition of clinical data. YT, JI, and YA analyzed and interpreted the data. HY, JI, and YA wrote the manuscript. All authors contributed to the article and approved the submitted version.

## Funding

Next-generation sequencing experiments were funded by the Nagoya University Graduate School of Medicine, under Grant [Japan Agency for Medical Research and Development (AMED)/16fk0108307h0402]. Other parts of this study were not funded by any institution.

## Conflict of Interest

The authors declare that the research was conducted in the absence of any commercial or financial relationships that could be construed as a potential conflict of interest.

## Publisher's Note

All claims expressed in this article are solely those of the authors and do not necessarily represent those of their affiliated organizations, or those of the publisher, the editors and the reviewers. Any product that may be evaluated in this article, or claim that may be made by its manufacturer, is not guaranteed or endorsed by the publisher.

## Abbreviations

UTI: urinary tract infection
EPA: emphysematous prostatic abscess
CT: computed tomography
MRI: magnetic resonance imaging
NGS: next-generation sequencing
MLST: multilocus sequencing typing
ST: sequence type
*kfuA*: an iron uptake system
*magA*: specific to K1 capsule serotype
*rmpA*: regulator of mucoid phenotype
*ybtS*: Yersiniabactin gene
*iroN*: Gene for the outer membrane receptor
FepA: of Fe^3+^-bound salmochelin
*mrkD*: type 3 fimbrial adhesin gene
hvKp: Hypervirulent *K. pneumoniae*
PLA: Pyogenic liver abscesses
cKp: Classical *K. pneumoniae*.
