# Supplementary figures and images for: Ruptured Emphysematous Prostatic Abscess Caused by K1-ST23 Hypervirulent Klebsiella pneumoniae Presenting as Brain Abscesses: A Case Report and Literature Review
Source: Front Med (Lausanne). 2022 Jan 3;8:768042. doi: 10.3389/fmed.2021.768042 (PMC8761798; doi:10.3389/fmed.2021.768042)

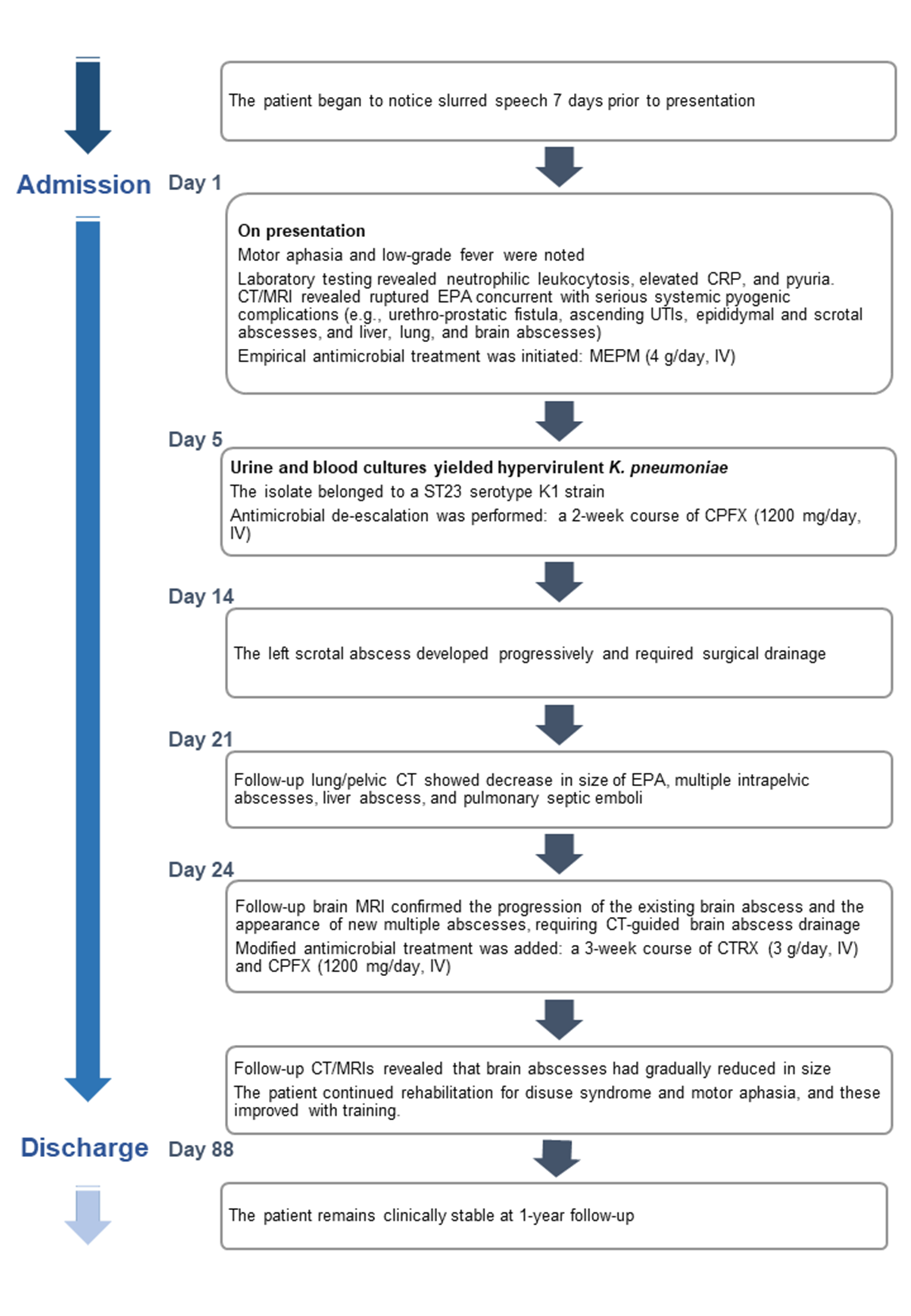

Supplement: Supplementary file 1 [file Image_1.TIF]
